# The Frustrated Host Response to *Legionella pneumophila* Is Bypassed by MyD88-Dependent Translation of Pro-inflammatory Cytokines

**DOI:** 10.1371/journal.ppat.1004229

**Published:** 2014-07-24

**Authors:** Seblewongel Asrat, Aisling S. Dugan, Ralph R. Isberg

**Affiliations:** 1 Howard Hughes Medical Institute, Boston, Massachusetts, United States of America; 2 Department of Molecular Biology and Microbiology, Tufts University School of Medicine, Boston, Massachusetts, United States of America; 3 Graduate Program in Molecular Microbiology, Sackler School of Graduate Biomedical Science, Tufts University School of Medicine, Boston, Massachusetts, United States of America; Yale University School of Medicine, United States of America

## Abstract

Many pathogens, particularly those that require their host for survival, have devised mechanisms to subvert the host immune response in order to survive and replicate intracellularly. *Legionella pneumophila*, the causative agent of Legionnaires' disease, promotes intracellular growth by translocating proteins into its host cytosol through its type IV protein secretion machinery. At least 5 of the bacterial translocated effectors interfere with the function of host cell elongation factors, blocking translation and causing the induction of a unique host cell transcriptional profile. In addition, *L. pneumophila* also interferes with translation initiation, by preventing cap-dependent translation in host cells. We demonstrate here that protein translation inhibition by *L. pneumophila* leads to a frustrated host MAP kinase response, where genes involved in the pathway are transcribed but fail to be translated due to the bacterium-induced protein synthesis inhibition. Surprisingly, few pro-inflammatory cytokines, such as IL-1α and IL-1β, bypass this inhibition and get synthesized in the presence of *Legionella* effectors. We show that the selective synthesis of these genes requires MyD88 signaling and takes place in both infected cells that harbor bacteria and neighboring bystander cells. Our findings offer a perspective of how host cells are able to cope with pathogen-encoded activities that disrupt normal cellular process and initiate a successful inflammatory response.

## Author Summary

Translation inhibition is a common virulence mechanism used by a number of pathogens (e.g. Diphtheria Toxin, Shiga Toxin and *Pseudomonas* Exotoxin A). It has been a mystery how host cells mount a pathogen-specific response and clear infection under conditions where protein synthesis is blocked by pathogens. Using *Legionella pneumophila* as a model, a bacterium that efficiently blocks the host protein translation machinery, we show here that the innate immune system has devised a mechanism to cope with translation inhibition by selectively synthesizing proteins that are required for inflammation.

## Introduction

The pathogen-associated molecular pattern (PAMP) hypothesis has been developed to explain how the innate immune system recognizes foreign microbial invaders. By this model, germline-encoded receptors recognize conserved foreign ligands associated with microbes, such as nucleic acids, lipopolysaccharide (LPS), peptidoglycan or flagellin to generate a response directed at clearing the microorganism [1], [2]. More recently, it has become clear that pattern recognition alone does not explain how multicellular organisms are able to differentiate virulent pathogens from harmless commensals and mount a response. It has been proposed that the host immune system can sense the presence of danger and respond to pathogen-encoded enzymatic activities that disrupt normal cellular processes. This mode of recognition, referred to as “effector triggered immunity” has been shown to play a significant role in pathogen clearance both in plants and mammalian cells [3], [4], [5], [6], [7], [8], [9], [10], [11], [12]. Such recognition may be sufficient to activate a host response, but because it occurs simultaneously with PAMP recognition, host cell detection of pathogens likely results from integrating the recognition of microbial patterns together with pathogen-specific activities.

*Legionella pneumophila*, the causative agent of Legionnaires' disease, promotes intracellular growth by translocating proteins into its host cytosol through its type IV (Dot/Icm) protein secretion machinery [13], [14], [15]. These translocated effectors serve various purposes, including recruitment of ER-derived membrane to the *Legionella* containing vacuole, inhibition of cell death pathways and manipulation of host lipid metabolism and regulatory pathways [16], [17], [18], [19], [20], [21]. Most importantly for the innate immune response, after contact with macrophages, the bacterium stimulates a pathogen-specific response that is the consequence of simultaneous recognition of PAMPs and pathogen-translocated proteins that results in a unique response to this microorganism [10].

*Legionella pneumophila* is a pathogen for a broad range of fresh water amoebae, which provide the natural environmental niche for the microorganism and the source of exposure for humans [22], [23]. After aspiration by a susceptible mammalian host, the bacterium is engulfed by alveolar macrophages in the lungs [24]. In cultured macrophages, *L. pneumophila* provokes signaling through various pattern-recognition receptors (PRRs), such as Toll-like receptors (TLRs) and cytosolic NOD-like receptors (NLRs) [9], [25], [26], [27], [28], [29], [30], [31]. This response is critical for clearance of the microorganism, because mouse mutants defective in these two responses succumb to lethal pneumonia [27].

Interestingly, macrophage challenge with wild type *L. pneumophila* (Dot/Icm^+^) triggers a unique transcriptional response in host cells compared to mutants that lack a functional type IV secretion system, supporting the model that there is a pathogen-specific response involved in innate immune recognition [9], [10], [11],[17],[32]. Microarray studies have identified many of these transcriptional targets as being genes controlled by the NF-κB and mitogen-associated protein kinases (MAPKs) transcriptional regulators [9], [17], including downstream dual specificity phosphatases (*Dusp1* and *Dusp2*), stress response genes (*Hsp70*, *Gadd45a*, *Egr1*) and pro-inflammatory cytokines and chemokines (*Il1α*, *Il1β*, *Tnfα*, *Il23a*, *Csf1*, *Csf2*) [9], [10], [11], .

It was recently demonstrated that the pathogen-specific response to *Legionella* is triggered by the action of *L. pneumophila* translocated effectors that interfere with host protein translation [10], [11]. Disruption of the host translation machinery serves as a second signal (in concert with signaling from PRRs) to constitute the full innate immune response against *Legionella pneumophila* [10]. The elimination of five of these effectors is sufficient to block this response, even though it is clear that they are part of a much larger pool of translocated substrates that impinge on host protein synthesis [10], [31]. These inhibitors (the products of the *lgt1*, *lgt2*, *lgt3*, *sidI*, and *sidL* genes) modify eukaryotic elongation factor eEF1A and eEF1Bγ of mammalian cells and block protein synthesis both *in vitro* and *in vivo* [10], [33], [34], [35].

In addition to blocking elongation, there is evidence that suggests wild type *Legionella* can also inhibit cap-dependent translation initiation [36]. Recognition of pathogenic *Legionella* leads to ubiquitination of the mTOR pathway, which in turn suppresses the eukaryotic initiation factor 4E (eIF4E) and prevents the synthesis of various genes [36]. This mode of translation inhibition was shown to induce translational biasing of host cells towards a more pro-inflammatory state [36]. However, it is currently not clear how host cells would be able to mount an inflammatory response when protein translation is blocked by *L. pneumophila* both at the initiation and elongation stages [9], [17]. It is likely that pattern-recognition would play a role under conditions of intoxication, but the mechanism by which this is regulated is also unclear.

A strong pro-inflammatory cytokine response is crucial for clearance of *Legionella pneumophila* [9], [31]. The importance of cytokines can be seen in IL-1α, IL-12, IFN-γ and TNF knockout mouse strains that show increased susceptibility to *L. pneumophila* infection [31], [37]. Moreover, patients treated with TNF-α blockers are at high risk of developing severe Legionnaires' disease [38]. Given the key role that this innate immune response plays in clearance of *L. pneumophila*, we examined how cytokines and other immune mediators are synthesized under conditions in which the bacterium effectively blocks the host protein translation machinery. We find that although protein synthesis inhibitors induce the transcriptional response and block the translation of most genes, pro-inflammatory cytokine genes can bypass this blockade in a fashion that requires the TLR-adaptor protein MyD88.

## Results

### *L. pneumophila* translation inhibitors induce a frustrated MAPK response

We hypothesized that *Legionella-*induced inflammatory gene transcription will be largely inconsequential, as the transcribed genes cannot be translated to proteins due to the bacterium-derived translation inhibitors. To test this hypothesis, we examined mammalian host protein synthesis predicted to be downstream of MAPK activation following exposure to *L. pneumophila*.

Bone marrow-derived macrophages challenged with wild type *L. pneumophila* showed phosphorylation of MAPK members shortly after exposure to the bacterium, with the activation kinetics being almost identical to previous observations ([9], [39], [40]; Figure S1). In the first hour after *L. pneumophila* challenge, activation of JNK and P38 was independent of the Icm/Dot system, consistent with phosphorylation being the result of Tlr engagement [9]. A second wave of activation was observed beginning at two hours after challenge. This was dependent on the presence of the *L. pneumophila* type IV secretion system, as the levels of MAPK phosphorylation decayed when macrophages were challenged with the *dotA3* mutant that lacks the Icm/Dot system (Figure S1). This two-wave activation, reflecting an early Tlr-dependent and a later *L. pneumophila*-specific response, mirrors our previous observations with NF-KB activation [41].

**Figure 1:**
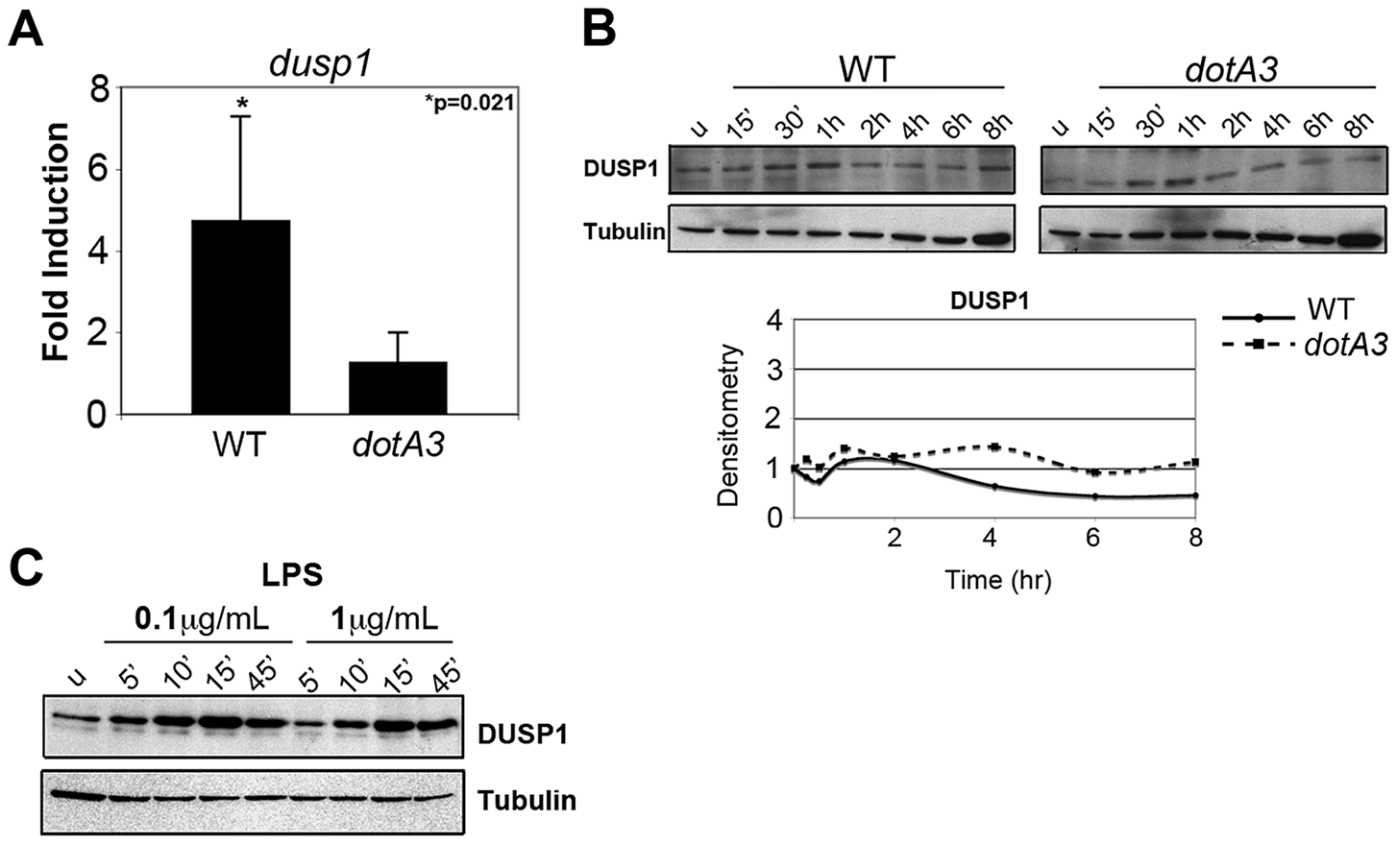
*L. pneumophila* translation inhibitors induce a frustrated MAPK response. (A) *Dusp1* transcript levels in A/J macrophages infected with wild type or *dotA3 L. pneumophila* for 4 hrs. Transcript levels were normalized to18S ribosomal RNA (18S) and graphed as a fold increase over uninfected controls. (B) Immunoblot analysis of DUSP-1 protein levels in A/J macrophages challenged with wild type or *dotA3* strains and (C) treated with LPS (0.1 µg/mL or 1 µg/mL) for indicated time points. Data are representative of at least three independent experiments.

Cultured macrophages challenged with wild type *L. pneumophila* transcriptionally activate a number of dual specificity phosphatase (DUSP) genes, dependent on an intact Icm/Dot system [17], [40]. Bone marrow-derived macrophages challenged with wild type *Legionella* for 4 hrs showed significant induction of the *Dusp1* transcript compared to *dotA3* infection (Figure 1A). The transcriptional induction was much higher (over 60 fold) in U937 human monocytes, the cell lines in which *dusp1* induction in response to wild type *Legionella* was first characterized (Figure S2A) [17]. This transcriptional response, however, was not accompanied by translation either in bone marrow-derived macrophages (Figure 1B) or U937 cells (Figure S2B) as DUSP-1 protein levels remained unchanged over the course of infection. Our inability to observe enhanced protein synthesis was not due to limitations with our detection system, because we observed a robust increase in DUSP-1 protein levels in response to the addition of LPS (Figure 1C). Presumably the multiple translocated substrates that inhibit translation elongation [10] frustrate the transcriptional response, preventing translation of induced genes. To test this hypothesis, we challenged cells with an *L. pneumophila* mutant missing five of the translation inhibitors (Δ*5*) to determine if the absence of these proteins could allow translation to proceed. Instead, we observed no transcriptional activation of the *Dusp1* gene in response to this mutant (Figure S2A). Therefore, induction of genes in the MAPK pathway and frustration of the response are tightly coupled.

### A subset of cytokine transcripts are selectively translated following *L. pneumophila* infection

We then asked if the response leading to transcriptional upregulation of pro-inflammatory cytokine genes was similarly affected by translational inhibition. To understand how cytokines are regulated in response to *L. pneumophila*, we measured the transcription and translation of selected pro-inflammatory cytokines in C57/Bl6 (B6) macrophages following *L. pneumophila* challenge. Flagellin deficient (*ΔflaA*) mutants were used in these experiments to avoid Caspase 1-dependent cell death downstream from NAIP5/NLRC4 recognition of flagellin by B6 macrophages [42], [43], [44].

**Figure 2:**
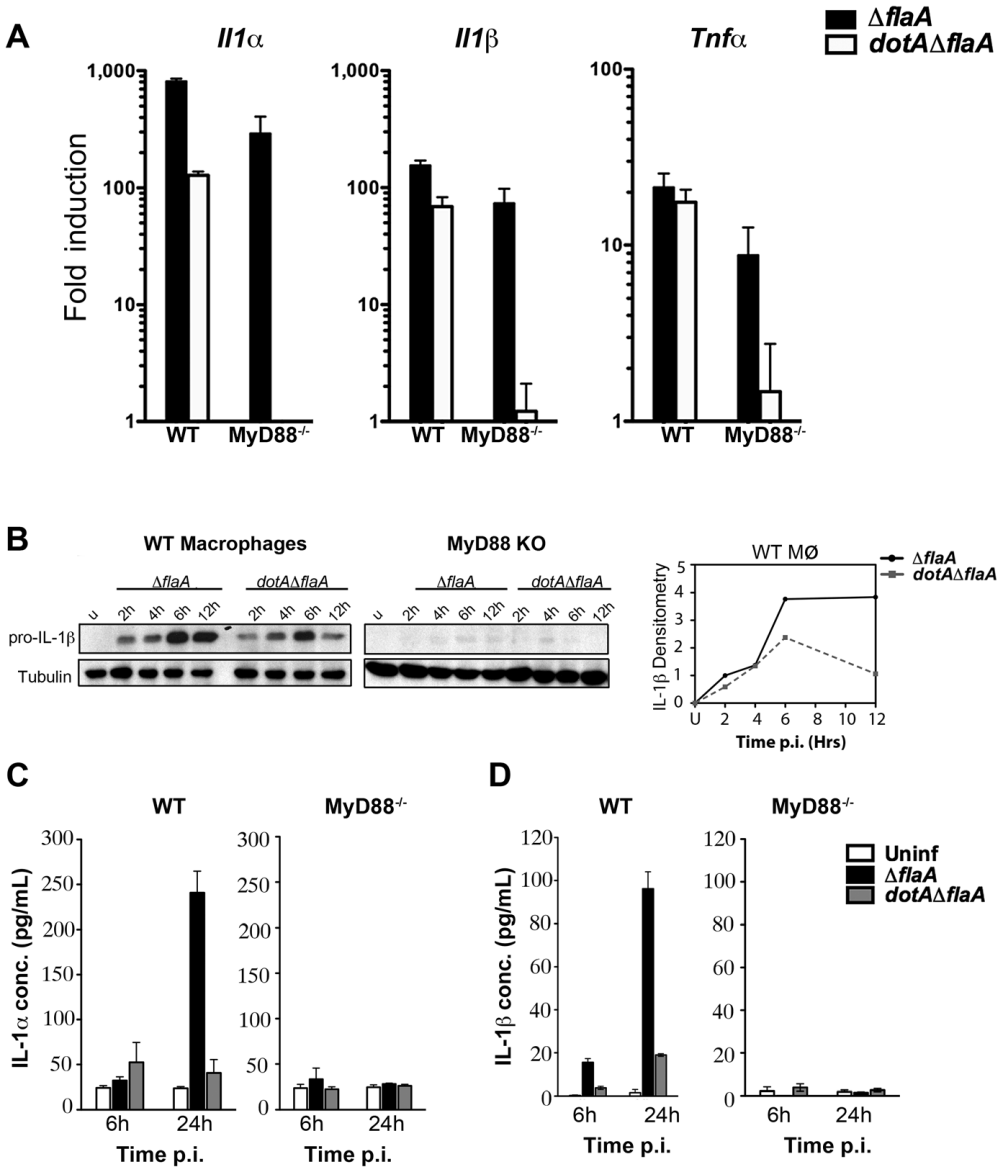
A subset of transcripts can bypass translation inhibition exerted by *L. pneumophila* effectors. (A) C57BL/6 wild type and MyD88^−/−^ macrophages were infected with indicated *L. pneumophila* strains at MOI-15. Cytokine and DUSP transcripts were analyzed at 6 hrs post infection by qRT-PCR. Results shown are pooled from at least four independent experiments and represent the mean fold induction and SEM of samples relative to uninfected controls. (B) Immunoblot analysis of IL-1β precursor in WT and MyD88^−/−^ macrophages infected with virulent *L. pneumophila* (*ΔflaA*) or avirulent mutant *dotAΔflaA* for indicated time points. Graphs on the right show densitometry of IL-1β normalized to tubulin. (C and D) WT and MyD88^−/−^ macrophages were challenged with indicated *L. pneumophila* strains for 6 and 24 hrs at MOI-15 and cytokine levels were measured in culture supernatants by ELISA. Data represent mean and SEM of samples from 3 independent experiments.

Infection of bone-marrow macrophages with virulent (Dot^+^) *L. pneumophila* induced *Il1α*, *Il1β* and *Tnfα* transcripts by 6 hrs post infection (Figure 2A). As previously reported, the cytokine response to Dot^+^ was comprised of MyD88-dependent signaling that is layered on top of MyD88-independent, effector mediated signaling (Figure 2A, black bars) [10]. The response to the avirulent, *dotA3* mutants on the other hand, was mostly dependent on MyD88 signaling (Figure 2A, white bars) [9].

To determine if the transcribed cytokine mRNAs were efficiently translated and secreted during infection, WT and MyD88^−/−^ macrophages were challenged with *L. pneumophila* and cytokine protein levels were measured by western blot and ELISA. Contrary to what we saw for DUSP-1, challenge with *L. pneumophila* Dot^+^ led to a significant increase in cell-associated pro-IL-1β levels after 4 hrs of infection (Figure 2B). IL-1α and IL-1β mature forms could also be detected in culture supernatants after 24 hrs (Figure 2C & 2D). Interestingly, challenge of macrophages with *L. pneumophila dotA3* mutants accumulated pro-IL-1β transiently, with steady state levels reduced by 12 hr post infection (Figure 2B densitometry), but this was not sufficient to induce the release of mature IL-1β. This is consistent with the hypothesis that in addition to TLR signaling, Icm/Dot translocated substrates are required for persistent pro-inflammatory cytokine activation and secretion [32]. Surprisingly, cytokine translation was severely diminished in MyD88^−/−^ macrophages in response to wild type *L. pneumophila* (Figure 2B) despite the presence of large amounts of transcripts (Figure 2A). Therefore, a MyD88-dependent signal appears necessary to bypass the translation block induced in response to wild type *L. pneumophila*.

### Intracellular cytokine accumulates in infected, translation-blocked macrophages

There is no clear model for why the presence of MyD88 allowed bypass of the translation block. Engagement of MyD88 on the host cell surface may lead to selective bypass of translation inhibition on a subset of transcripts. Alternatively, translation of cytokine transcripts could largely occur in neighboring uninfected cells that have not been directly injected with *L. pneumophila* translocated proteins, but which have been activated by bacterial fragments liberated by infected cells. We therefore asked if the observed cytokine translation was derived from neighboring bystander cells.

**Figure 3:**
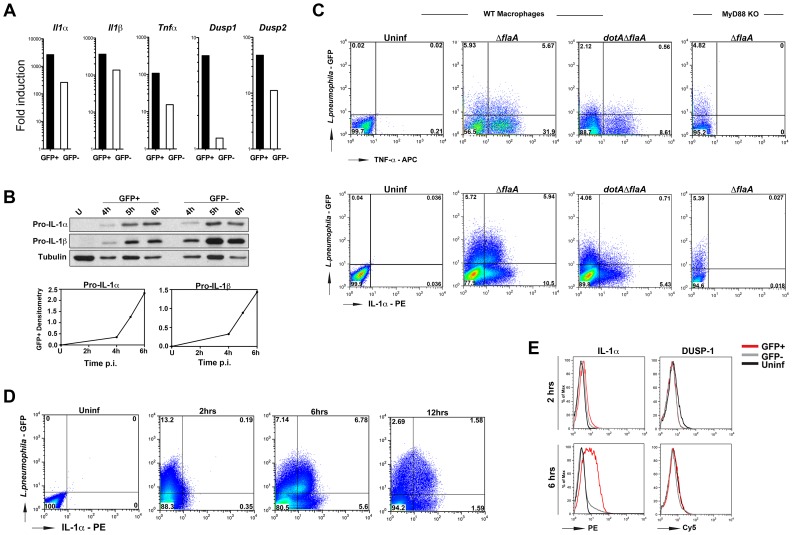
Cytokines are produced from both infected and bystander macrophages. (A) B6 macrophages were challenged with *ΔflaA*-GFP at MOI-15 for 4 hrs and sorted by Flow Cytometry. Cytokine and *Dusp* transcripts were measured in both GFP^+^ and GFP^−^ population by qRT-PCR. (B) B6 macrophages were infected with *ΔflaA*-GFP at MOI-15 for indicated times and levels of IL-1β were measured by Western blot. Bottom graphs indicate densitometry of IL-1β in GFP^+^ cells normalized to tubulin. (C) WT and MyD88^−/−^ macrophages were infected with indicated *L. pneumophila* strains at MOI-10 and intracellular cytokine levels were measured by flow cytometry. Top panels show TNF levels at 14 hrs post infection. To prevent secretion of TNF, cells were treated with Golgiplug (Brefeldin A) for 5 hrs before samples were collected. Bottom panels show intracellular IL-1α levels at 6 hrs post infection. Data shown are representative of at least 4 independent experiments. (D) Time course analysis of intracellular IL-1α levels in *ΔflaA*-GFP infected macrophages and (E) Comparison of intracellular IL-1α and DUSP1 levels in B6 macrophages infected with *ΔflaA*-GFP for 2 and 6 hrs. Infected and uninfected (bystander) cells were gated based on GFP signal and protein levels were compared to control macrophages that were left untreated. Red lines indicate GFP^+^ population; grey lines indicate GFP^−^ population and black lines show untreated macrophages.

B6 macrophages were challenged with *L. pneumophila-*GFP strains and macrophages harboring bacteria were sorted from uninfected bystanders. Cytokine transcripts (Figure 3A) and protein levels (Figure 3B) were measured in each population by qRT-PCR and Western blots. To ensure accumulation of TNF-α protein, cells were treated with GolgiPlug (Brefeldin A; Materials and Methods) to prevent secretion of this cytokine. No such treatment was necessary for IL-1α and IL-1β, which accumulate as precursors via an alternate secretion pathway [45].

**Figure 4:**
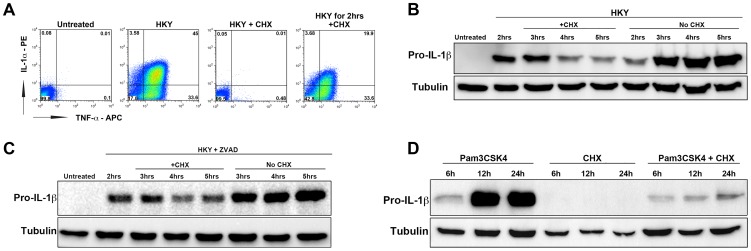
The elongation inhibitor cycloheximide blocks cytokine translation independent of cell death. (A) B6 macrophages were either left untreated (1^st^ box), treated with heat killed *Yersinia* at MOI = 50 to induce cytokine expression (2^nd^ box), treated with HKY MOI = 50 and 10 µg/mL cycloheximide (CHX) (3^rd^ box) or treated with HKY MOI = 50 for 2 hrs followed by addition of 10 µg/mL CHX (4^th^ box). X-axis represents intracellular TNF-α levels and Y-axis represents intracellular IL-1α levels. (B) Macrophages were pre-stimulated with heat killed *Yersinia* at MOI = 50 for 2 hrs. Cells were then treated with either 1 µg/mL cycloheximide or left untreated (HKY alone). IL-1β protein levels were measured by Western blot at the indicated time points. (C) B6 macrophages were pre-stimulated with heat killed *Yersinia* at MOI = 50 in the presence of Pan-Caspase inhibitor (Z-VAD-FMK) for 2 hrs and treated with either 0.5 µg/mL cycloheximide or left untreated. IL-1β protein levels were measured by Western blot for the next 3 hrs. (D) Macrophages were treated with 2 µg/mL Pam3CSK4 for 2 hrs in the presence of Z-VAD-FMK. CHX (0.5 µg/mL) was added to cells at 2 hrs post Pam3CSK4 treatment and IL-1β protein levels were measured at 6, 12 or 24 hrs. Data is representative of at least 3 independent experiments.

Relative to cells that had never been exposed to bacteria, challenge of macrophages with *L. pneumophila* resulted in high levels of *I1α*, *Il1β*, *Tnfα* and *Dusp2* transcripts in both infected (GFP^+^) and neighboring uninfected (GFP^−^) populations by 4 hrs post infection, (Figure 3A). Depending on the cytokine, the amount of transcription in the bystander cells varied from 10% −30% of that observed in the cells harboring bacteria. *Dusp1* on the other hand, was mainly transcribed in GFP^+^ cells (Figure 3A). More importantly, despite the presence of the translocated protein synthesis inhibitors, macrophages harboring bacteria were able to produce high levels of pro-IL-1α and pro-IL-1β (Figure 3B, GFP^+^ cells). The kinetics of pro-IL-1α and pro-IL-1β production in infected cells indicated that there was enhanced accumulation of these proteins between 4–6 hrs post-infection (Figure 3B, bottom panel). We will show that during this time window, *L. pneumophila* translation inhibitors effectively block most protein synthesis in infected cells (below, Figure 4B).

The presence of persistent cytokine synthesis in infected cells was confirmed by intracellular cytokine staining. B6 WT and MyD88^−/−^ macrophages were challenged with *L. pneumophila*-GFP strains and intracellular cytokine levels were measured by flow cytometry. TNF-α was produced by both macrophages bearing bacteria (GFP^+^) and bystander cells (GFP^−^) after challenge with *L. pneumophila Δfla*, while the *dotAΔfla* strain induced much lower levels of this cytokine (Figure 3C, top panel 2^nd^ and 3^rd^ boxes). Cells harboring bacteria (GFP^+^) were a significant source of IL-1α (Figure 3C, bottom panel, 2^nd^ box). Approximately 50% of the cells harboring bacteria showed detectable accumulation of IL-1α (Figure 3C, bottom panels, 2^nd^ box), while bacteria were associated with approximately 33% of the IL-1α-producing cells. Consistent with Figure 2B, translation of IL-1α and TNF-α were both dependent on MyD88 signaling, and accumulation of IL-1a in infected cells was dependent on the presence of the Icm/Dot translocator (Figure 3C, two rightmost boxes in each panel).

Time course analysis of intracellular IL-1α levels using flow cytometry confirmed that the highest level of IL-1α accumulation occurred between 2–6 hrs post infection in the GFP^+^ population (Figure 3D and 3E). During this time period, the number of cells bearing bacteria that accumulated IL-1α increased from 1% of this population to approximately 50% (Figure 3D). DUSP-1 protein levels on the other hand, remain unchanged between 2–6 hrs (Figure 3E).

### Cytokine response to chemical inhibitors of protein translation

The two signals that are received by mammalian cells during *L. pneumophila* infection (1^st^ signal from TLR activation and 2^nd^ signal from protein translation inhibition) synergize to induce the full cytokine response against the bacterium. It was previously reported that pharmacological inhibitors of host protein translation induce transcription of various stress response genes and cytokines such as IL-6, IL-23, IL-α and IL-1β [10], [31]. We wanted to confirm that translation and secretion of these cytokines could always bypass translation inhibition using the protein synthesis inhibitor cycloheximide (CHX). CHX interferes with protein translation elongation by binding to the E-site of the 60S ribosomal subunit and preventing tRNA translocation [46].

Macrophages were treated with heat-killed *Yersinia* (HKY) to induce TLR signaling, together with 10 µg/mL of cycloheximide. Addition of the chemical inhibitor at the same time as HKY led to a complete inhibition of TNF-α and IL-1α production in bone-marrow macrophages (Figure 4A). Contrary to what we observed during *L. pneumophila* infection, addition of CHX dampened the signal received from TLR stimulation (Figure 4 A,B). Surprisingly, even at low concentrations of CHX that permit significant levels of protein translation (Figure S3), CHX was still able to inhibit IL-1β translation (Fig. 4B).

To rule out the possibility that the reduction in IL-1β levels during CHX treatment was due to cell death, we lowered the CHX dose to 0.5 µg/mL (inhibits less than 50% of total host protein synthesis) (Figure S3) and also incubated the cells with the apoptotic inhibitor Z-VAD-FMK (pan-caspase inhibitor). CHX was still able to inhibit IL-1β under these conditions, although it was clear that increased survival of cells was accompanied by higher accumulation of HKY-induced cytokine (Figure 4C).

MyD88-dependent stimulation of mouse macrophages in response to *L. pneumophila* primarily occurs via Toll-like receptor 2 (TLR2) [29]. A recent report was able to reconstruct the cytokine induction seen during *L. pneumophila* infection by using the synthetic TLR-2 ligand Pam3CSK4 in combination with Exotoxin A (Exo A), a toxin from *Pseudomonas aeruginosa* that interferes with translation elongation [31]. Based on this observation, we wanted to determine if specific activation of TLR2 is what leads to cytokine translation in the presence of protein synthesis inhibitors. Macrophages were treated with the TLR2 agonist Pam3CSK4 and pro-IL-1β levels were measured in the presence or absence of the protein synthesis inhibitor cycloheximide (CHX). Drug treatment after addition of the TLR2 agonist led to a large reduction in pro-IL-1β levels (Figure 4D). We also observed a failure to hyperstimulate pro-IL-1b in the presence of another protein elongation inhibitor, puromycin (Figure S4). Similar results were obtained when macrophages were stimulated with another TLR2 agonist lipoteichoic acid (LTA), or a TLR4 agonist LPS, followed by addition of CHX (data not shown). This indicates that the selective synthesis of cytokines may result from host cells sensing a specific mode of protein synthesis inhibition. It is also possible that the selective synthesis of pro-inflammatory cytokines is triggered by a block in translation initiation [36] instead of translation elongation, which would explain why the elongation inhibitors cycloheximide or puromycin were not able to induce the response.

### Translation of IL-1 takes place in the presence of highly depressed host cell translation

We considered two possible explanations for how cytokines were translated in the presence of the bacterium-derived protein synthesis inhibitors: (1) translation inhibition by *L. pneumophila* is not efficient, allowing most of the cytokine to be synthesized prior to a complete block; or (2) the host preferentially translates a subset of genes after protein synthesis is shutdown by pathogens.

To distinguish between these possibilities, relative levels of protein synthesis were measured in bone-marrow macrophages at various times following *L. pneumophila* challenge, using an immunofluorescence readout. B6 macrophages were challenged with *L. pneumophila flaA^−^*-GFP at MOI = 10 for 2 hrs and translation of proteins was measured by incorporation of a methionine analog (L-azidohomoalanine, AHA) for an additional 4 hrs. Incorporated AHA was detected by reaction with a fluorescent-labeled phosphine reagent (phosphine-APC), which covalently links to the azido-functional group on AHA (Staudinger ligation reaction) [47].

**Figure 5:**
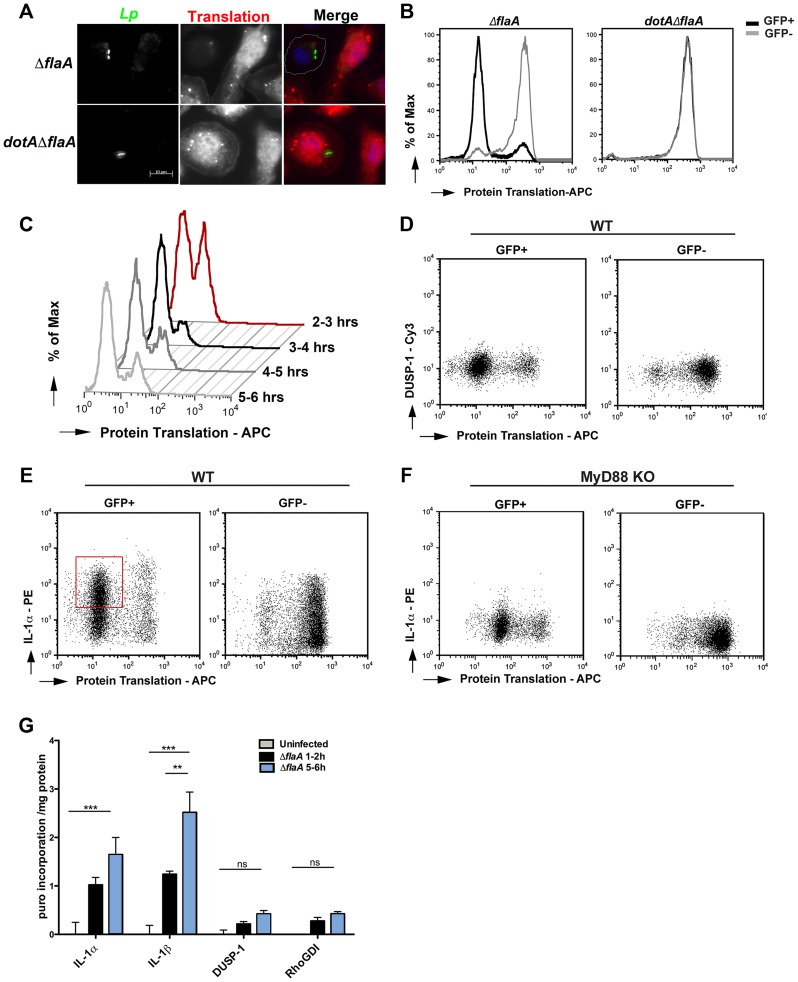
Translation of IL-1 takes place in the presence of *L. pneumophila* elongation inhibitors. (A) B6 macrophages were infected with indicated *L. pneumophila* strains at MOI = 10 for 2 hrs and a methionine analog, L-azidohomoalanine (AHA, 50 µM) was incorporated into newly synthesized proteins for 4 hrs. Cells were fixed, permeabilized and the incorporated analog was detected by an APC conjugated phosphine and fluorescence microscopy. Nuclei were stained with Hoechst 33342 (blue) (B) Macrophages were infected with indicated *L. pneumophila* strains at MOI = 10 for 2 hrs and L-azidohomoalanine (AHA, 50 µM) was added to cells for additional 4 hrs. Protein translation was quantified in infected macrophages (GFP^+^) and uninfected bystanders (GFP^−^) by staining with APC-labeled phosphine and flow cytometry. Experiment was performed three times. (C) B6 macrophages were infected with Dot^+^ *L. pneumophila* for 2 hrs and AHA was added for 1 hr intervals. Cells were fixed after each time point and protein translation was quantified by APC-conjugated phosphine and flow cytometry. Translation of DUSP1 (D) or IL-1α (E) was monitored in wild type macrophages infected with Δ*flaA* for 6 hrs by co-staining AHA with Cy3 conjugated DUSP1 antibody or PE-conjugated IL-1α antibody. (F) Translation of IL-1α in MyD88-deficient macrophages was determined by co-staining AHA with PE-conjugated IL-1α antibody. Dot plots show GFP^+^ (left column) and GFP^−^ (right column) gated cells. (G) Kinetics of IL-1α, IL-1β, DUSP-1 and RhoGDI translation was quantified in Dot+ infected macrophages using puromycin incorporation. B6 macrophages were infected with Dot+ *L. pneumophila* and 10 µg/mL of puromycin was added either between 1–2 hrs or between 5–6 hrs post infection. Cells were washed, lysed and incubated on plates coated with anti- IL-1α, IL-1β, DUSP-1 and RhoGDI antibodies. Incorporation of puromycin in the indicated samples was monitored by anti-puromycin antibody and HRP-conjugated secondary antibody. Data represent absorbance at 450 normalized to total protein levels. Values for uninfected controls were subtracted from each sample to determine the increase in puromycin incorporation upon infection. Each bar represents mean and SEM of triplicate samples.

Protein translation was significantly inhibited in macrophages harboring *L. pneumophila* (Δ*flaA*) compared to bacteria lacking the Icm/Dot system (Figure 5A). Furthermore, in macrophage cultures incubated with *L. pneumophila*, the macrophages harboring *L. pneumophila* showed selective protein synthesis interference, while the majority of the uninfected cells showed efficient incorporation of the amino acid analog (Figure 5B; compare GFP^+^ to GFP^−^ population). To determine the time point at which the protein synthesis inhibitors fully shut down global protein translation, pulse-chase experiments were performed in which the methionine analog (AHA) was added for 1 hr intervals starting at 2 hrs post infection (Figure 5C). Between 2–3 hrs post-infection, approximately 40% of the cells harboring *L. pneumophila* were found in the population that has high levels of protein synthesis. Between 3–4 hrs post infection, we observed a major shift where almost 90% of cells harboring Δ*flaA* were found in the population having highly depressed protein synthesis. Later time points showed no further blockage in translation, perhaps reflecting the fact that there is a small fraction of wild type bacteria that fail to form replication compartments [48]. This population is predicted to show no significant translocation via the Icm/Dot system and should fail to inhibit protein synthesis (seen in ∼10% of the macrophage population).

To determine if translation-blocked cells could still produce cytokines, macrophage monolayers were challenged with *L. pneumophila-*GFP^+^ and protein synthesis was monitored by addition of AHA. The cells were then probed for IL-1α accumulation by immunofluorescence and flow analysis. In the infected GFP^+^ population of macrophages, the majority of cells that accumulated IL-1α show evidence of an almost complete shutdown of protein translation (Figure 5E; red box). In the absence of MyD88 signaling, both the infected and uninfected populations showed little IL-1α accumulation (Figure 5F). In contrast to IL-1α, there was no DUSP-1 accumulation after *L. pneumophila* challenge of macrophages (Figure 5D). This confirmed our main hypothesis that there is translation of selected cytokine genes when protein synthesis is inhibited by *L. pneumophila* and this bypass requires MyD88 signaling.

### The increase in IL-1α and IL-1β protein levels is due to enhanced protein synthesis

We have shown that pro-IL-1α and pro-IL-1β accumulate in cells that harbor *L. pneumophila* between 4–6 hrs post infection (Figure 3B), despite a significant block in protein translation (Figure 5C & E). To confirm that the accumulation we observe in infected cells was due to newly synthesized cytokines over the course of infection, we took advantage of another protein translation assay, SunSET. This assay uses puromycin incorporation into growing polypeptide chains to monitor active protein synthesis [49]. We modified this assay to measure the amount of puromycin incorporated into our protein of interest during a 1-hour pulse period. Accordingly, macrophages were challenged with *L. pneumophila* for increasing lengths of time, cells were labeled for one hour with 10 µg/mL of puromycin, lysed, and individual proteins were immobilized in assay wells using specific antibodies (Materials and Methods). An ELISA was then used to determine the amount of puromycin incorporated in the immobilized proteins (Figure 5G). Consistent with our intracellular cytokine staining and Western blot data (Figure 3), the highest levels of IL-1α and IL-1b synthesis were detected between 5–6 hrs post-infection. On the other hand, no significant puromycin incorporation was detected for DUSP-1 and RhoGDI proteins, confirming that there is selective synthesis of few genes after *L. pneumophila* challenge.

### Translation inhibition acts in concert with TLR signaling to generate the full cytokine response

**Figure 6:**
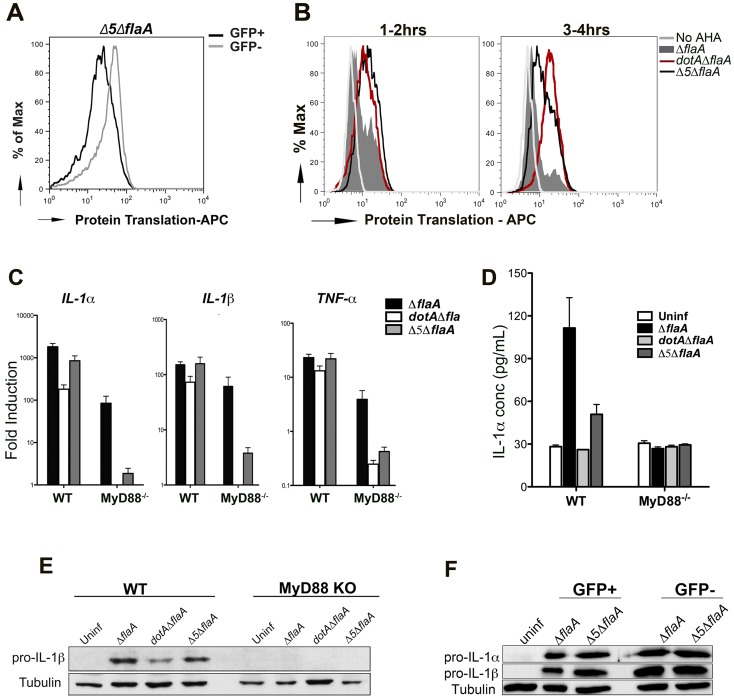
Production of the pro-inflammatory cytokines IL-1α and IL-1β is independent of the five translocated protein synthesis inhibitors (A) B6 macrophages were infected with Δ5Δ*fla*-GFP+ and protein translation was measured between 2–6 hrs post infection by incorporation of the methionine analog AHA. Translation (incorporation of AHA) was compared between infected cells (GFP+, black line) and uninfected bystanders (GFP−, grey line). (B) B6 macrophages were infected with GFP expressing Dot+, Dot− and Δ5 strains of *L. pneumophila* and protein synthesis inhibition was compared between these strains by incorporation of AHA between 1–2 hrs (left graph) or 3–4 hrs (right graph). Graphs show translation in infected cells (GFP+). Cells incubated in the absence of the methionine analog (No AHA) were used as a negative control to show baseline staining. (C) WT and MyD88^−/−^ macrophages were infected with the indicated strains at MOI-15 and cytokine transcripts were analyzed at 6 hrs post infection by qRT-PCR. Data represent the mean fold induction and SEM of samples relative to uninfected controls. (D) ELISA measurement of IL-1α secretion from WT and MyD88^−/−^ macrophages infected with the indicated strains for 24 hrs. Data represent mean and SEM of triplicate samples. (E) Immunoblot analysis of pro-IL-1β in WT and MyD88^−/−^ macrophages challenged with the indicated *L. pneumophila* strains for 6 hrs. (F) B6 macrophages were challenged with *ΔflaA*-GFP or *Δ*5*Δfla* at MOI-15 for 6 hrs and sorted by Flow Cytometry. Pro-IL-1β levels were measured in both GFP+ and GFP− population by westernblot.

To determine the role that *L. pneumophila* translation inhibitors play in modulating host cytokine synthesis, we used a mutant that lacks the 5 Icm/Dot translocated substrates known to block host protein synthesis (Δ*5* mutant) [10]. The level of protein synthesis was first measured using AHA incorporation (Figure 6A, B) and puromycin incorporation (Figure S5) in macrophages infected with Δ*5*Δ*flaA-*GFP^+^. Compared to Dot^+^ strain (Figure 6B & Figure S5), there was an increase in active protein translation in cells that were infected with the Δ*5* mutant, although the cells showed lower levels of protein synthesis than Dot− infected cells (Figure 6B) or the uninfected population (Figure 6A; compare GFP^+^ to GFP^−^ populations). This is consistent with the hypothesis that in addition to Icm/Dot translocated substrates that act on translation elongation, infection with virulent *L. pneumophila* also blocks translation initiation [36].

It had been previously reported that in the absence of most known pathways of pattern recognition (MyD88^−/−^ Nod1^−/−^ Nod2^−/−^ macrophages), the cytokine transcriptional response to *L. pneumophila* was primarily due to the presence of the translocated protein synthesis inhibitors [10], [11]. Using macrophages that are only defective for MyD88, this dependence on the translation inhibitors could be clearly observed for the *Il1α*, *Il1β* and *Tnfα* transcripts (Figure 6C, MyD88^−/−^, gray vs. black bars). Consistent with our previous data, the transcriptional response in MyD88 knockout macrophages was unproductive, with no evidence that these highly induced transcripts are translated (Figure 6E).

In the case of wild type macrophages, Dot^+^ and Δ*5* infections induced comparable levels of MyD88-dependent cytokine transcription and translation (Figure 6C and E), and this could be observed in macrophages that were sorted by flow cytometry, as well (Figure 6F). This result is in contrast with macrophages lacking MyD88 signaling, in which it is clear that there is protein synthesis inhibitor-dependent induction of cytokine transcripts (Figure 6C), but this induction produces no apparent cytokine translation products. Interestingly, unlike what we see for *dotA* mutants, infection with Δ*5* was still able to induce secretion of mature IL-1α at 24 hrs after infection [31], even though it was to a lesser extent than wild type (Figure 6D).

Therefore, although the protein synthesis inhibitors are responsible for the transcriptional response that occurs in the absence of pattern recognition receptors, the production of cytokine proteins associated with infections by fully virulent strains is not dependent on these translocated substrates.

### MyD88-mediated bypass of translation inhibition of the *il1β* gene is independent of mRNA stability

**Figure 7:**
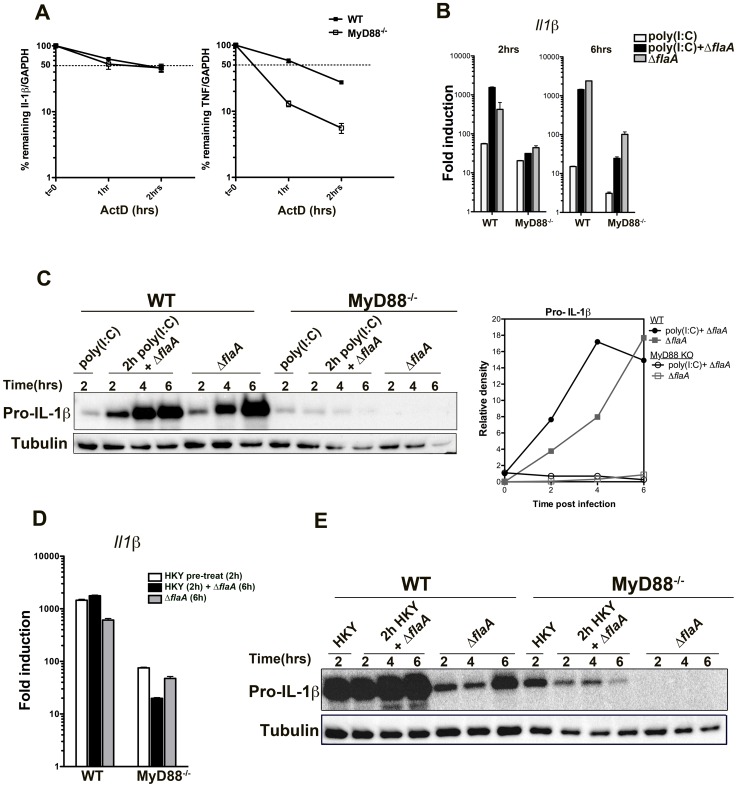
Stability of Il-1β mRNA or pre-activation of the NF-kB pathway is not sufficient to induce translation bypass in MyD88^−/−^ macrophages. (A)WT and MyD88 deficient bone-marrow macrophages were challenged with Δ*flaA* for 2.5 hrs and transcription was blocked by addition of 10 µg/mL Actinomycin D. The percentage of remaining *Il1β* and *Tnfα* transcripts was measured by qRT-PCR after 1 or 2 hrs post actinomycin D treatment. Data represent mean and SEM of samples relative to GAPDH. (B) WT and MyD88^−/−^ macrophages were pre-treated with 50 µg/mL poly(I∶C) for 2 hrs to induce NF-kB activation, after which cells were either challenged with Dot+ *Legionella* or left untreated (poly(I∶C) only). Macrophages that were not pre-stimulated with poly(I∶C) but were challenged with Dot+ were included for comparison. RNA was collected after 2 and 6 hrs of infection and *Il1β* transcript levels were analyzed by qRT-PCR. (C) B6 WT and MyD88-deficient macrophages were pre-treated with poly(I∶C) for 2 hrs or left untreated. Cells were then challenged with Dot+ *Legionella* for additional 2, 4 and 6 hrs and production of pro-IL-1β was examined by Western blot. Densitometry of the blot is shown on the right. (D) WT and MyD88^−/−^ macrophages were pre-treated with HKY (MOI = 100) for 2 hrs or left untreated after which cells were challenged with Dot+ *L. pneumophila*. Induction of *Il1β* transcript was determined after 6 hrs of infection. (E) Pro-IL-1β synthesis was also examined in these cells after 2, 4 and 6 hrs of challenge.

Cytokine expression is regulated at various stages, including transcription, post-transcriptional processing, translation and secretion. One of the main regulatory steps for IL-1 and TNF production is their transcript stability, which is controlled by their AU-rich elements (ARE) in their 3′-noncoding regions and by various ARE-binding proteins [50], [51]. To determine if lack of cytokine translation in MyD88^−/−^ macrophages was due to mRNA instability, the half-life of *Il1β* and *Tnfα* transcripts were compared in WT and MyD88^−/−^ macrophages after *L. pneumophila* challenge. Macrophages were first infected with *L. pneumophila* for 2.5 hrs, actinomycin D was added to the medium to block further transcription, and the amount of transcript remaining was measured by qRT-PCR at various time points after the addition of the drug. *Tnfα* mRNA was highly unstable in the absence of MyD88 when compared to MyD88^+/+^ macrophages (Figure 7A). However, *Il1β* transcripts were relatively stable in the absence of MyD88, and the amount of mRNA remaining after 2 hrs was similar to control macrophages (Figure 7A). This indicates that the translation inhibition bypass of *Il1β* was independent of mRNA stability (Figure 7). In the case of *tnfα*, however, transcript stabilization via a MyD88-dependent signal may play a role in bypassing translation inhibition.

### Pre-activation of NF-kB in MyD88-deficient macrophages is not sufficient to bypass translation inhibition

Another possible explanation for why few pro-inflammatory cytokines, such as IL-1α and IL-1β, bypass translation inhibition could be mRNA abundance. A recent study has shown that these cytokines are the most abundant transcripts within macrophages after challenge with *L. pneumophila* [36]. Although there was significant transcription of these genes in MyD88^−/−^ macrophages, the total mRNA abundance of IL-1α, IL-1β and TNF was still significantly lower compared to wild type macrophages (Figure 2). To address if mRNA abundance plays a role in bypass of translation inhibition during *Legionella* infection, WT and MyD88^−/−^ macrophages were pre-activated with the TLR3 agonist poly(I∶C) for 2 hrs to induce NF-kB signaling. We also used heat-killed *Yersinia pseudotuberculosis* (HKY) to activate NF-kB via TLR4.

2 hr pre-stimulation with 50 µg/mL poly(I∶C) was sufficient to trigger IL-1β transcription (Figure 7B, left graph, white bars) both in wild type and MyD88^−/−^ macrophages. Pre-stimulation with poly(I∶C) followed by *L. pneumophila* infection increased *Il1β* transcription initially (after 2 hrs of infection) in wild type macrophages compared to cells that were untreated (Figure 7B, compare black bars with grey bars). Surprisingly, this was reversed by 6 hrs post infection and cells that were pre-treated with poly(I∶C) down regulated their *Il1β* transcription (Figure 7B, right graph). This was more pronounced in MyD88^−/−^ macrophages (Figure 7B, compare black and grey bars). We observed a very similar phenomenon when cells were pre-treated with heat-killed *Yersinia* followed by *Legionella* infection (Figure 7D).

As expected, pro-IL-1β translation was robust in wild type macrophages that were pre-treated with poly(I∶C) or with HKY followed by *Legionella* infection (Figure 7C and 7E). Interestingly, pro-IL-1β was detected by Western blots in MyD88^−/−^ macrophages after pre-stimulation with poly(I∶C) but the protein level was reduced when the pre-activated cells were challenged with *L. pneumophila* (Figure 7C). MyD88^−/−^ macrophages that were infected with *Legionella* alone on the other hand, showed little detectable translation (Figure 7C) despite having higher levels of *Il1β* transcripts (Figure 7B, right graph, compare black and grey bars). This phenotype was more obvious during pre-treatment with HKY. 2 hr HKY pre-stimulation led to a robust pro-IL-1β translation initially, which was significantly reduced when cells were challenged with *L. pneumophila* (Figure 7E). Therefore, in the absence of MyD88 signaling, macrophages were unable to overcome the translation inhibition induced by *Legionella pneumophila* even in the presence of external stimuli.

## Discussion

Inhibition of protein translation is a common virulence mechanism used by many viruses and bacteria. In this study, we showed that host cells have evolved mechanisms to cope with translation inhibition by selectively translating a subset of cytokine genes, including pro-inflammatory cytokines such as IL-1α and IL-1β in response to *L. pneumophila* challenge. The ability to bypass *L. pneumophila* translational inhibition is an important determinant of host protection, as mice defective in the IL-1α/IL-1β response and humans exposed to TNF-α inhibitors are highly susceptible to *L. pneumophila* infection [31], [38].

*L. pneumophila* challenge of bone-marrow macrophages leads to a dramatic reduction in global protein translation (Figure 5A). The bacterium interferes with protein translation both at the initiation step [36] and elongation step [10]. It has been shown previously that this inhibition triggers the transcription of various stress response genes including NF-kB- and MAPK-regulated genes, heat shock proteins and pro-inflammatory cytokines and chemokines [9], [10], [11]. We show here that *L. pneumophila* translocated effectors prevent the translation of these genes, resulting in a “frustrated response,” in which there is accumulation of transcripts but no increase in protein levels. A subset of cytokine genes, and potentially other genes that have not been identified yet, are insensitive to this inhibition and get translated in cells that show the highest level of protein synthesis inhibition.

We observed bypass of translational inhibition as an orderly series of events resulting in the accumulation of IL-1α in cells harboring bacteria as well as in bystanders, followed by release of the cytokine into culture supernatants. Initial transcriptional induction and translation of IL-1a occurred independently of the Icm/Dot system, and was associated with TLR-signaling, consistent with the TLR-dependent activation of the NF-κB response known to occur at early time points after *L. pneumophila* challenge [41]. This was followed by persistent accumulation of pro-IL-1α protein in a process that required both the presence of the Icm/Dot system and MyD88-dependent signaling, indicating collaborative signaling between the two pathways. Surprisingly, accumulation of pro-IL-1α was equally robust in both cells harboring bacteria and in bystander cells, in spite of the translocated protein synthesis inhibitors that are deposited by *L. pneumophila*. This observation is particularly striking, in that time points showing the strongest inhibition of protein synthesis also resulted in the fastest rate of pro-IL-1α accumulation, arguing that there is selective ribosomal loading of cytokine transcripts in intoxicated cells. In the absence of MyD88 signaling, no such bypass could be observed in either infected or uninfected cells, either because translation requires the extremely high levels of transcription that occur in the presence of MyD88, or there is pattern recognition-dependent bypass of translational inhibition. Accumulation of pro-IL-1α was then followed by its release, which required both the Icm/Dot system and MyD88.

It is conceivable that the *L. pneumophila* translational inhibitors could be responsible for the induction of pro-IL-1α. Arguing against this model is the fact that a strain that lacks 5 of the known translation inhibitors (Δ5) still induced considerable pro-IL-α accumulation (Figure 6E, F) [31]. The accumulation of cytokine in response to this strain could have resulted from residual translation inhibition that was observed, but it should be noted that MAPK and NF-κB activation resulting from macrophage challenge by this strain is due to a pattern recognition response and is not due to Icm/Dot signals [11]. The initial MyD88-dependent activation that occurs after contact with *L. pneumophila* may be amplified by unknown Icm/Dot-signals or due to inhibition of translation initiation [36]. A recent study proposed that infection of macrophages with virulent *L. pneumophila* strains (both Dot+ and Δ5) leads to downregulation of mTOR activity, which is sufficient to suppress cap-dependent protein translation initiation [36]. The second signal that is required for amplifying pattern-recognition could be generated from such translational suppression, and could be the trigger for induction of pro-IL-1α.

Challenge of macrophages that lack MyD88 with *L. pneumophila* induces cytokine gene transcription, but the transcribed genes fail to be translated. In the absence of MyD88, therefore, the protein translation inhibition takes on global dimensions. The MyD88-dependent bypass of the translation inhibition was independent of transcript stability in the case of *Il1β* transcript, which is a known strategy for post-transcriptional regulation of cytokines [52], [53]. This surprising result indicates that there may be a previously unrecognized MyD88-dependent signaling pathway that mediates post-transcriptional regulation of cytokine transcripts. It has previously been reported that separate transcriptional and translational signals are required for IL-1β expression [54]. Although it is not clear what these translational signals could be, it is possible that MyD88-dependent signals could result in either enhanced ribosome loading, or could regulate translation via action at the 3′ or 5′ untranslated regions. Alternatively, the role of MyD88 could be totally passive, and merely a consequence of enhancing expression of cytokine gene transcripts. Although *L. pneumophila* infection causes a large induction of cytokine transcription in the absence of MyD88, these levels are still lower than what is seen when pattern recognition is intact (Figure 2A). This added boost in cytokine gene transcription by MyD88 may be sufficient to push the concentrations of these transcripts above the minimum threshold necessary to support selective translation of these genes under conditions of intoxication. Consistent with this model are previous results from nanostring analysis of macrophage transcripts that are induced in response to *L. pneumophila* challenge [36]. In this work, it is argued that the primary determinant of translation in cells challenged with *L. pneumophila* is the relative abundance of a particular transcript. Translation was most likely to occur from transcripts that were the most abundantly expressed after bacterial challenge [36].

A similar phenomenon to that reported here has been observed in the model organism *C. elegans* upon infection with *Pseudomonas aeruginosa*. *C. elegans* intestinal cells endocytose *P. aeruginosa* Exotoxin A, which shuts down protein translation by inhibiting elongation factor 2 (EF2) [8]. This inhibition leads to the selective translation of ZIP-2, which is required for activation of defense pathways and pathogen clearance [7]. It was proposed that the 5′ UTR of zip-2, which contains several untranslated ORFs (uORFs), was required for the selective translation. Even so, there is no explanation for how ribosome loading and translation can selectively occur in this transcript. Interestingly, there are a few other examples from mammalian cells and yeast, where protein translation inhibition leads to selective translation of few genes that have uORFs at their 5′ UTR. The mammalian stress response transcription factor ATF4 and the yeast transcription factor GCN4 respond similarly to amino acid starvation and protein synthesis inhibition [55], [56]. The 5′ UTR or 3′ UTR of cytokines could potentially be functioning the same way to allow selective protein translation when initiation and/or elongation is blocked by *Legionella pneumophila*.

Interestingly, pharmacological inhibitors of host protein translation induce transcription of various stress response genes, including pro-inflammatory cytokines such as *Il6*, *Il23*, *Il1α* and *Il1β* [10], [11], [31]. Secretion of these genes can take place when the highly conserved host elongation machinery is targeted by toxins such as *P. aeruginosa* Exotoxin A or *Corynebacterium diphtheriae* encoded diptheria toxins [10], [31]. *Diphtheria* toxin and *Pseudomonas* ExoA inhibit eukaryotic elongation factor similar to the mechanism used by *L. pneumophila* effectors. They modify elongation factor 2 (EF2) of eukaryotic cells by ADP-ribosylation, which has been shown to trigger a strong host immune response [31], [57]. This suggests the presence of a conserved surveillance mechanism the host uses to detect and respond to inhibition of the translation elongation machinery.

A previous study has shown that the cytokine induction seen during *L. pneumophila* can be mimicked by the addition of *P. aeruginosa* Exo A in combination with the synthetic PAMP ligand Pam3Csk4 [31]. It seems likely that macrophages are able to selectively bypass the translation block of this toxin in a fashion that is similar to that described in *C. elegans*. Interestingly, we cannot reproduce this result using a variety of concentrations of the protein synthesis inhibitor cycloheximide, which instead reduces pro-inflammatory cytokine production in response to PAMP challenge. The mechanism by which cycloheximide inhibits protein synthesis is sufficiently different from these toxins to explain why we see differences in the innate immune response against CHX. CHX binds to the E-site of the 60S ribosomal subunit and freezes all translating ribosomes [46].The RNA/ribosome complex remains stabilized and does not dissociate, a phenomenon that may not be perceived as danger by eukaryotic cells. In contrast, both *L. pneumophila* and ExoA interfere with elongation factor function. There may be a set of modified elongation factors in the host cell that resist action of these effectors, or there may be a population that is sequestered from modification, allowing them to selectively act on cytokine transcripts. In either case, there must be some special property to the cytokine transcript that allows this selective utilization of these active elongation factors. Future work will focus on the nature of these transcripts that allows bypass of translation inhibition.

## Materials and Methods

### Ethics statement

This study was carried out in accordance with the recommendation in the Guide for Care and Use of Laboratory Animals of the National Institutes of Health. The Institutional Animal Care and Use Committee of Tufts University approved all animal procedures. Our approved protocol number is B2013-18. The animal work, which is limited to the isolation of macrophages, does not involve any procedures of infections of live animals.

### Bacteria and culturing media

*L. pneumophila* strains Lp02 (referred to as WT) and Lp03 (referred to as *dotA3*) are streptomycin-resistant restriction-defective thymidine auxotrophs derived from *L. pneumophila* Philadelphia-1 (Lp01) (Table 1; [58]). The Δ5 and Δ5.Δ*flaA* strains were kindly provided by Zhao-Qing Luo (Purdue University) [10], [11], [43]. *ΔflaA*-GFP^+^, *dotAΔflaA*-GFP^+^, Δ5.Δ*flaA*-GFP^+^ carry GFP on an isopropyl-β-D-thiogalactopyranoside (IPTG)–inducible, Cm resistant plasmid (Table 1; [17], [59]). Solid medium containing buffered charcoal yeast extract (BCYE) and ACES-buffered yeast extract (AYE) broth culture medium supplemented with 100 µg/mL thymidine were used to maintain *L. pneumophila* strains [60], [61]. Strains containing the pGFP plasmid were maintained on BCYE plates containing 100 µg/mL thymidine and 5 µg/mL chloramphenicol and grown in AYE containing 100 µg/mL thymidine, 5 µg/mL chloramphenicol and 1 mM IPTG [17].

**Table 1:** Plasmids and strains used in this study.

Plasmids/Strains	Genotype/relevant characteristics	Reference
pGFP	oriRSF101*cm^R^ptac::*GFP^+^	[17], [59]
p*ΔflaA*	pSR47S*ΔflaA*	[63]
WT	Lp02, Philadelphia-1, *thyArpsLhsdR*	[58]
*dotA3*	Lp02 *dotA03* (Lp03)	[58]
*ΔflaA*	Lp02*ΔflaA* (allelic exchange using p*ΔflaA*)	[43]
*dotA.ΔflaA*	Lp03*ΔflaA* (allelic exchange using p*ΔflaA*)	[63]
*Δ5*	Missing 5 IDTS required for host protein translation inhibition	[10]
*Δ5.ΔflaA*	Lp02 background	[11]
*ΔflaA*-GFP	*ΔflaA* containing pGFP	This study
*dotA.ΔflaA*-GFP	*dotA3.ΔflaA* containing pGFP	This study
Δ5.Δ*flaA*-GFP	Δ5.Δ*flaA* containing pGFP	This study

### Eukaryotic cell culture

Bone marrow-derived macrophages (BMDMs) were isolated from the femurs of mice and allowed to proliferate as described [60], [61]. C57BL/6 *myd88^−/−^* femurs were kindly provided by Tanja Petnicki-Ocwieja in the laboratory of Linden Hu (Tufts Medical Center). BMDMs were differentiated for 7 days in RPMI containing 30% L-cell supernatant, 10% FBS, 2 mM L-glutamine and 1× Pen Strep (100 U/mL penicillin, 100 µg/mL streptomycin). Cells were lifted and either re-plated for experiments or quick-frozen for later use in FBS and 10% DMSO.

U937 cells (ATCC) were grown in RPMI supplemented with 10% FBS and 1 mM L- glutamine. For differentiation, cells were treated with 10 ng/ml 12-tetradecanoyl phorbol 13-acetate (TPA) for 48 hrs. For *L. pneumophila* infections, U937 cells were plated in fresh media without TPA and infections were carried out 12–16 hours after plating.

### Immunoblotting

To evaluate protein expression in host cells, C57BL/6 bone marrow-derived macrophages were plated in medium supplemented with 200 µg/mL of thymidine. Cells were challenged with *L. pneumophila* at the desired MOI, subjected to centrifugation at 1000×g for 5 min and incubated at 37° for the noted time periods. Lysates were collected using 2× SDS Laemmli sample buffer (0.125 M Tric-Cl pH 6.8, 4% SDS, 20% glycerol, 10% beta-mercaptoethanol, 0.01% bromophenol blue). Proteins were electroblotted to PVDF membranes, blocked in milk and analyzed by immunoprobing.

For phospho-specific antibodies, blots were washed of all milk and incubated overnight with 1∶1000 phospho-p38 or phospho-JNK (Cell Signaling) in 5% BSA in phosphate buffered saline (PBS). Rabbit anti-DUSP1 (MKP1 V-15, Santa Cruz) and mouse anti-tubulin (sigma) antibodies were diluted to 1∶200 and 1∶7,500, respectively, in 5% milk in TBST. Goat anti-IL-1α and Goat anti-IL-1β antibodies (R&D systems, AF-400-NA & AF-401-NA) were diluted to 1∶500 in 5% milk in TBST.

### Heat Killed Yersinia, LPS, Pam3CSK4, poly(I∶C) treatment

For preparation of Heat-Killed Yersinia, wild type *Y. pseudotuberculosis* strains were grown overnight at 26° in Luria-Bertani (LB) broth. Overnight cultures were heat killed at 60° for 30–60 min and aliquots were frozen at −80° until use.

Macrophages were stimulated with HKY (MOI = 50), LPS (Sigma, 0.1 µg/mL or 1 µg/mL) and Pam3CSK4 (Invitrogen, 2 µg/mL), poly(I∶C) (InvivoGen, 50 µg/mL) for the desired time points. Cells were washed 3× with PBS and lysed in 2× SDS Laemmli sample buffer.

### Quantitative RT-PCR

RNA was extracted from mammalian cells using RNAeasy kit (Qiagen). To determine the amount of a particular transcript, a one step, RNA-to-C_t_ kit (Applied Biosystems) was used according to manufacturer's instructions. Primers used for transcript analysis were as follows: human *Dusp1* (5′ TTTGAGGGTCACTACCAG and 3′ CCGCTTCGTAGTAGAG), mouse *Dusp1* (5′GGATATGAAGCGTTTTCGGCT and 3′ ACGGACTGTCACGTCTTAGG), mouse *Il1α* (5′GCACCTTACACCTACCAGAGT and 3′ TGCAGGTCATTTAACCAAGTGG), mouse *Il1β* (5′ GCAACTGTTCCTGAACTCAACT and 3′ ATCTTTTGGGGTCCGTCAACT), mouse *Tnf-α* ( *5′* GCACCACCATCAAGGACTCAA and 3′ GCTTAAGTGACCTCGGAGCT), mouse 18S ribosomal RNA (5′ CGCCGCTAGAGGTGAAATTCT and 3′ GCTTTCGTAAACGGTTCTTCA).

### Determination of secreted cytokines

Macrophages were plated in 24 well tissue culture plates (2.5×10^5^ per well) and challenged with *L. pneumophila* for either 6 hrs or 24 hrs. Supernatants were collected from each sample and 50 µL was used for ELISA. Mouse IL-1α and IL-1β Platinum ELISA (eBiosciences) was used to measure cytokine levels according to the manufacturers manual.

### Intracellular cytokine staining

Intracellular cytokine staining was performed as described before [62] with modifications. Differentiated macrophages (∼1×10^7^cells/plate) were washed and challenged with *L. pneumophila* GFP^+^ strains in RPMI containing 200 µg/mL thymidine, 5 µg/mL Chloramphenicol and 1 mM IPTG. For measuring TNF production, infections were carried out at MOI-3 and MOI-10 for 9 hrs followed by Golgiplug incubation (BD Bioscience, 1 µL/mL) for additional 5 hrs. For IL-1α and IL-1β, infections were carried out for 6 hrs. Cells were harvested with 10 mL cold PBS, washed twice with FACS buffer (PBS+0.5%BSA+0.05%NaN), incubated with Fc Block (clone 2.4G2) for 20 minutes and fixed with 2% paraformaldehyde overnight at 4°. Macrophage were permeabilized with Perm/Wash buffer (BD Bioscience) on ice for 20 minutes and stained with Alexa Fluor 647-conjugated anti-mouse TNF (BioLegend, clone ALF 161), phycoerythrin(PE)-conjugated anti-mouse IL-1α (BioLegend, clone MP6-XT22), PE-conjugated anti-mouse IL-1β (eBioscience, clone NJTEN3), Rabbit anti-DUSP1 (MKP1 V-15, Santa Cruz) and Goat anti-Rabbit Cy5 (Invitrogen) for 40 minutes. Stained cells were analyzed by BD LSR II flow cytometer.

### Translation, pulse labeling and quantification

To detect whole cell translation during defined timepoints, C57BL/6 bone marrow-derived macrophages were challenged with *L. pneumophila*-GFP^+^ at MOI-10 for 2 hrs, followed by labeling cells at various timepoints with 50 µM of L-azidohomoalanine (AHA) (Invitrogen) added to the culture medium. Cells were incubated for 1 hr (time course experiments) or for 4 hrs (IL-1α co-staining experiments) to allow incorporation of AHA into growing polypeptide chains. At the end of each incubation period, cells were washed with PBS, fixed with 4% paraformaldehyde for 15–20 min and left overnight in PBS. Incorporation of AHA was monitored by a Biotin- or APC-conjugated phosphine reagent (Pierce) and nuclei were stained with Hoechst 33342 (Molecular Probes). For flow cytometry, fixed cells were blocked with 1× BSA/PBS for 30 min at RT and 100 µM of APC-phosphine was added. Cells were incubated at 37° for 2–3 hrs and excess dye was removed by washing with 0.5% Tween-20/PBS. For IL-1α co-staining, PE conjugated anti-mouse IL-1α (BioLegend, clone MP6-XT22) was added to cells on ice for 30 min. Cells were analyzed by BD LSR II flow cytometer or by BD FACScalibur.

### Puromycin incorporation (SunSET assay) and ELISA

SunSET assay was used to determine the kinetics of protein translation over time [49] with modifications. Macrophages were plated in 6 well plates and infected with Δ*flaA* for the desired time points. 10 µg/mL of puromycin (Sigma) was added to cells for either 15 min or 1 hr. Macrophages were washed and lysed with IP Wash/Lysis buffer (Pierce) in the presence of protease inhibitors (Roche). Lysates were incubated on ice for 20 min, centrifuged at 13,000 rpm for 5 min and the supernatants were used for ELISA. The protein concentration in each sample was determined by Bradford assay.

ELISA plates were prepared by coating 96 well Nunc MaxiSorp plates with the desired antibody. Polyclonal Goat anti-IL-1α and IL-1β (R&D systems), Rabbit anti-DUSP-1 (Santa Cruz) and Rabbit anti-RhoGDI (Santa Cruz) antibodies were diluted in Carbonate/Bicarbonate buffer (PH = 9.6) to 10 µg/mL and 100 µL was used per well. Plates were incubated overnight at 4°. The following day, plates were brought to room temperature and blocked with 0.5% BSA/PBS for 1 hr. Cell lysates that have incorporated puromycin were incubated on the ELISA plates for 2 hrs, washed with 0.05% Tween-20/PBS three times and incubated with monoclonal mouse-anti-Puromycin (12D10, Millipore) for 1 hr. Unbound antibody was washed exhaustively with 0.05% Tween-20/PBS and plates were incubated with Donkey anti-mouse-HRP secondary antibody. Unbound antibody was washed again with Tween-20/PBS four times and 100 µL of HRP substrate (TMB solution) was added to each well for 5–10 mins. The reaction was stopped by adding 100 µL of stop-solution (2N H_2_SO_4_), and absorbance was measured at 450 nm.

## Supporting Information

Figure S1 ***L. pneumophila*** **induces an early TLR-dependent and a later Icm/Dot-dependent activation of MAPK members.** A/J macrophages were infected with wild type *L. pneumophila* or *dotA3* mutant for indicated time points. Cell lysates were blotted for phosphorylated forms of JNK (p-JNK) and p38 (p-p38). Lower graphs show densitometry of p-JNK and p-p38 normalized to tubulin. Data are representative of at least three independent experiments.
(TIF)

Figure S2 **Δ5 strains fails to induce transcription of DUSP-1 in U937 human monocytes.** (A) U937 cells were challenged with wild type *L. pneumophila*, *dotA* or Δ5 mutants for 4 hrs and RNA was isolated from cells. *dusp1* transcript levels were normalized to the housekeeping genes hydroxymethylbilane synthase (HMBS) and graphed as a fold increase over uninfected controls. (B) A time course analysis of DUSP1 protein levels in U937 cells infected with wild type, *dotA* or Δ5 strain of *L. pneumophila*.
(TIF)

Figure S3 **Low concentrations of CHX allow partial protein translation to take place.** B6 macrophages were either left untreated or treated with the indicated concentrations of cycloheximide for 1 hr and the methionine analog, L-azidohomoalanine (AHA, 50 µM) was incorporated into newly synthesized proteins for an additional hour. The incorporated analog was detected by an APC-conjugated phosphine and fluorescence microscopy. Bottom panel shows protein translation in macrophages that were infected with Dot+ *L. pneumophila* and AHA added between 5–6 hrs post infection.
(TIF)

Figure S4 **Both cycloheximide and puromycin block IL-1β hyperstimulation.** Macrophages were treated with 2 µg/mL Pam3CSK4 for 2 hrs. Cells were then treated with either cycloheximide (0.5 µg/mL) or puromycin for additional 3 hrs and pro- IL-1β protein levels were measured by western blot.
(TIF)

Figure S5 **Δ5 mutants show partial inhibition of protein synthesis.** B6 macrophages were infected with Δ*flaA*, *dotA*Δ*flaA* and Δ*5*Δ*flaA* for 5 hrs and 10 µg/mL of puromycin was added between 5–6 hrs poi. Cells were fixed, permeabilized and incorporated puromycin was detected by anti-puromycin antibody (12D10) and fluorescence microscopy.
(TIF)
