# Supplementary figures and images for: The Frustrated Host Response to Legionella pneumophila Is Bypassed by MyD88-Dependent Translation of Pro-inflammatory Cytokines
Source: PLoS Pathog. 2014 Jul 24;10(7):e1004229. doi: 10.1371/journal.ppat.1004229 (PMC4110041; doi:10.1371/journal.ppat.1004229)

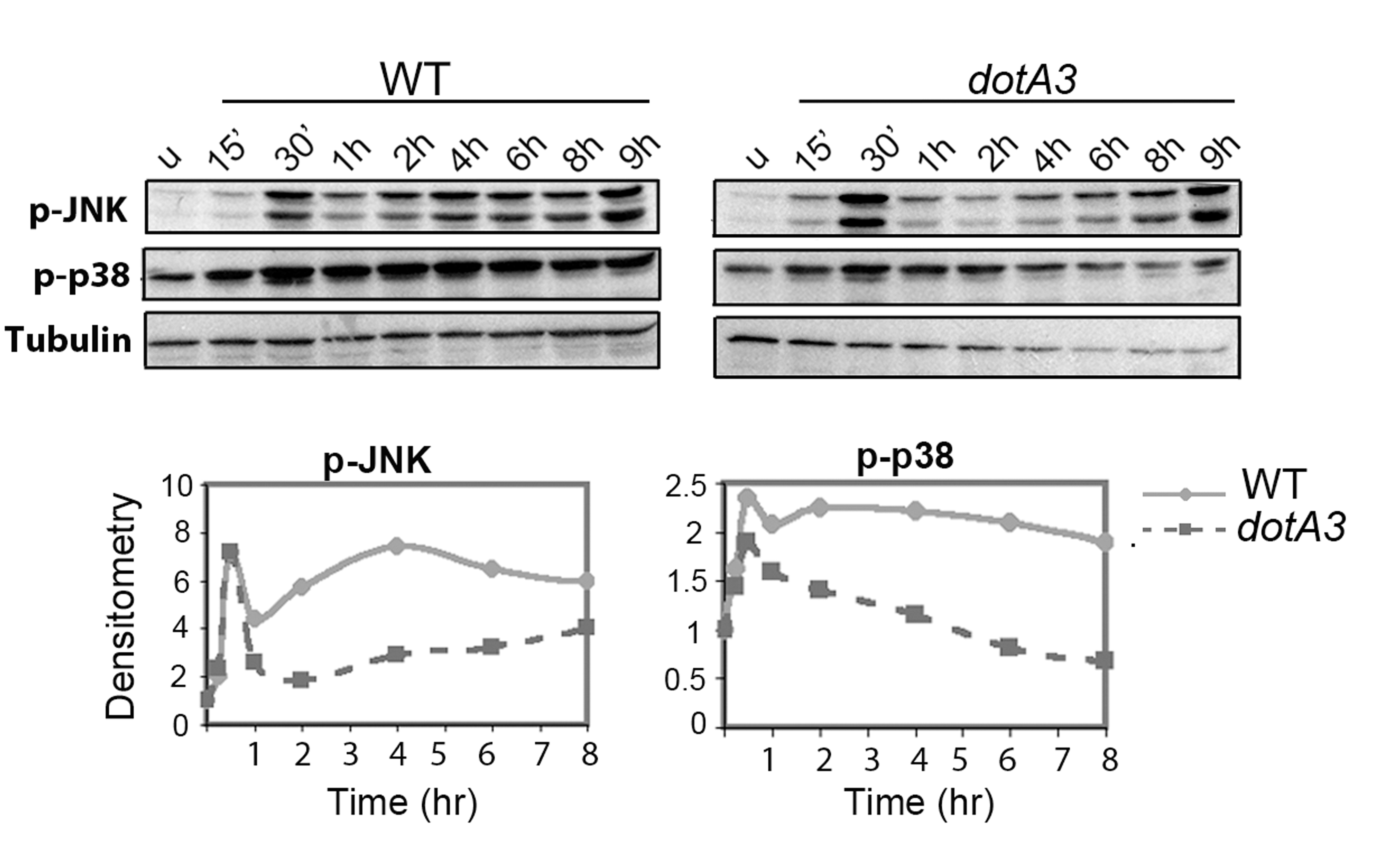

Supplement: Figure S1 — L. pneumophila induces an early TLR-dependent and a later Icm/Dot-dependent activation of MAPK members. A/J macrophages were infected with wild type L. pneumophila or dotA3 mutant for indicated time points. Cell lysates were blotted for phosphorylated forms of JNK (p-JNK) and p38 (p-p38). Lower graphs show densitometry of p-JNK and p-p38 normalized to tubulin. Data are representative of at least three independent experiments. (TIF) [file ppat.1004229.s001.tif]

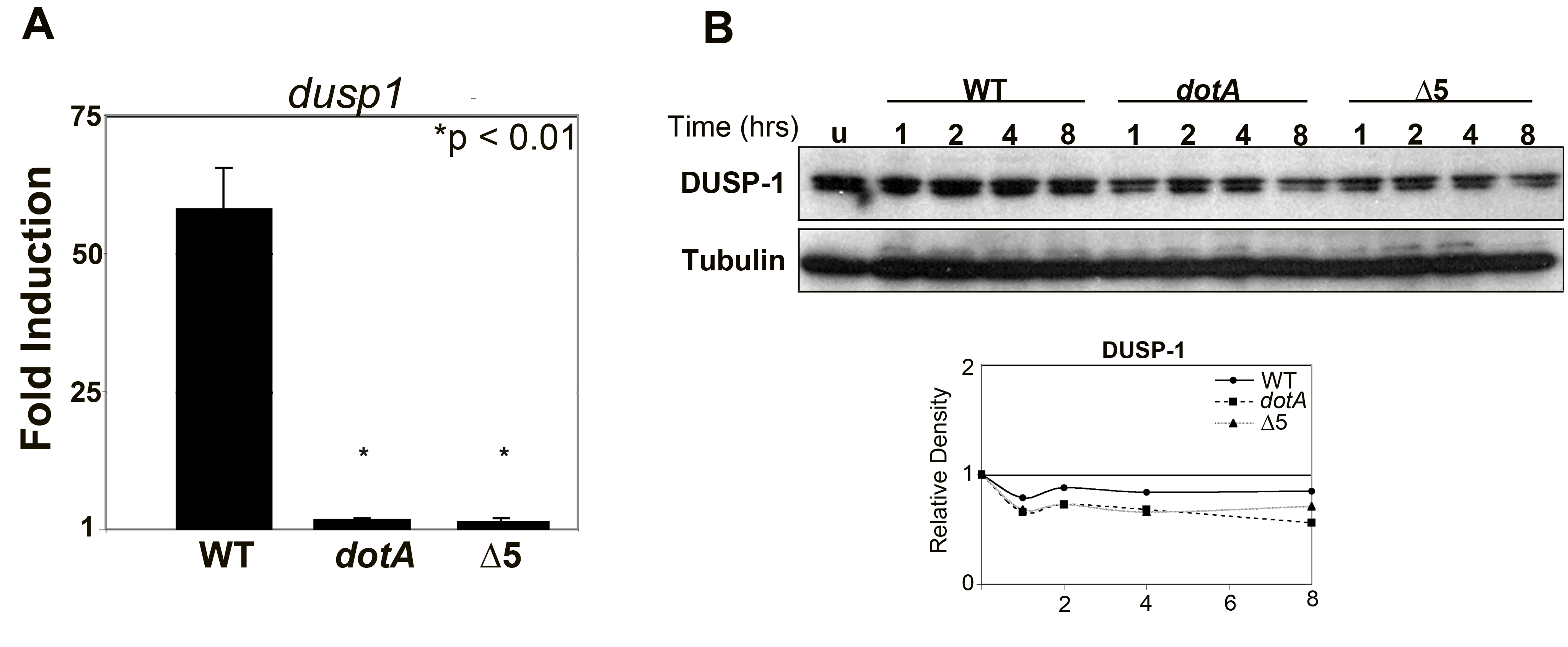

Supplement: Figure S2 — Δ5 strains fails to induce transcription of DUSP-1 in U937 human monocytes. (A) U937 cells were challenged with wild type L. pneumophila, dotA or Δ5 mutants for 4 hrs and RNA was isolated from cells. dusp1 transcript levels were normalized to the housekeeping genes hydroxymethylbilane synthase (HMBS) and graphed as a fold increase over uninfected controls. (B) A time course analysis of DUSP1 protein levels in U937 cells infected with wild type, dotA or Δ5 strain of L. pneumophila. (TIF) [file ppat.1004229.s002.tif]

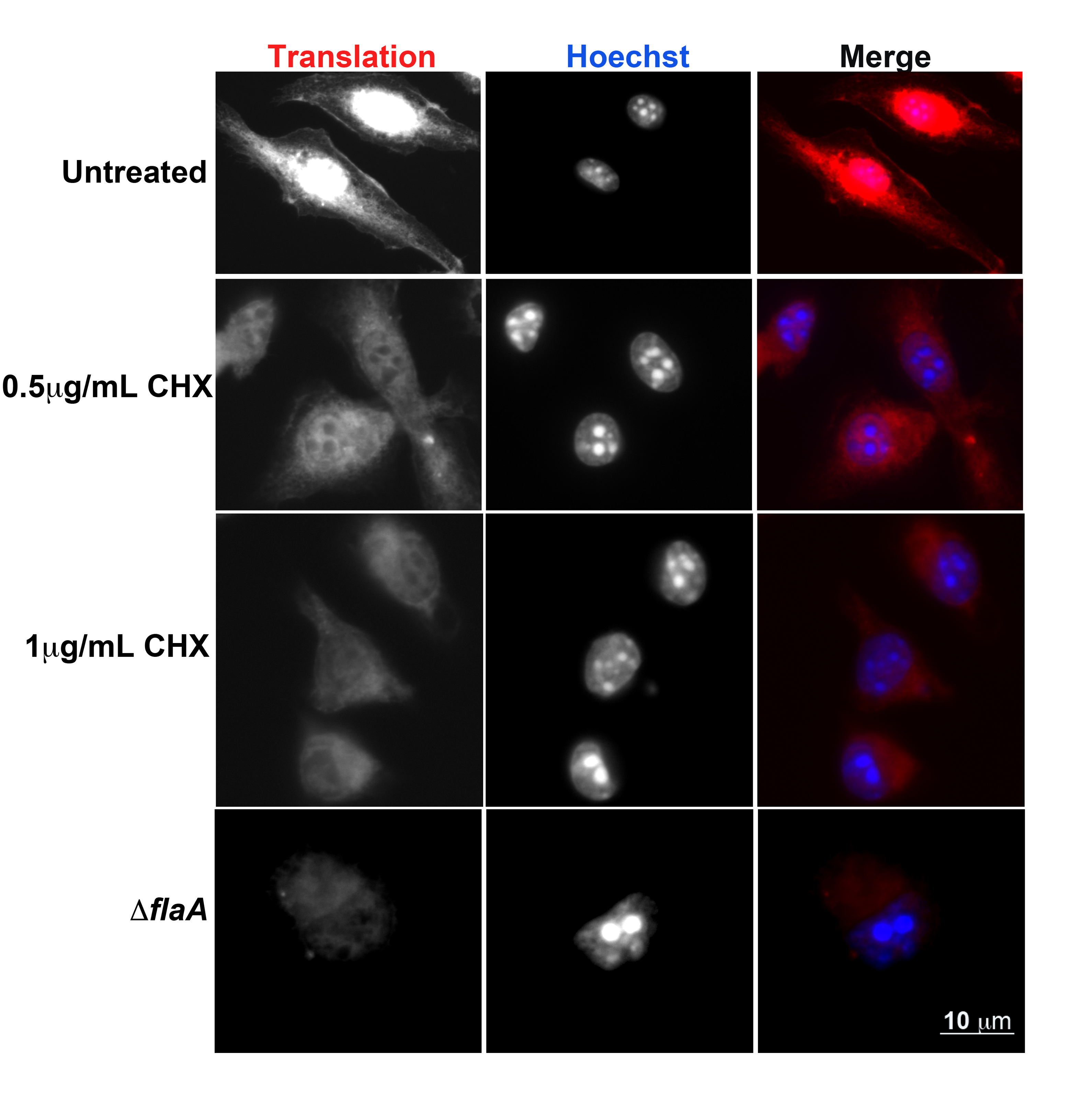

Supplement: Figure S3 — Low concentrations of CHX allow partial protein translation to take place. B6 macrophages were either left untreated or treated with the indicated concentrations of cycloheximide for 1 hr and the methionine analog, L-azidohomoalanine (AHA, 50 µM) was incorporated into newly synthesized proteins for an additional hour. The incorporated analog was detected by an APC-conjugated phosphine and fluorescence microscopy. Bottom panel shows protein translation in macrophages that were infected with Dot+ L. pneumophila and AHA added between 5–6 hrs post infection. (TIF) [file ppat.1004229.s003.tif]

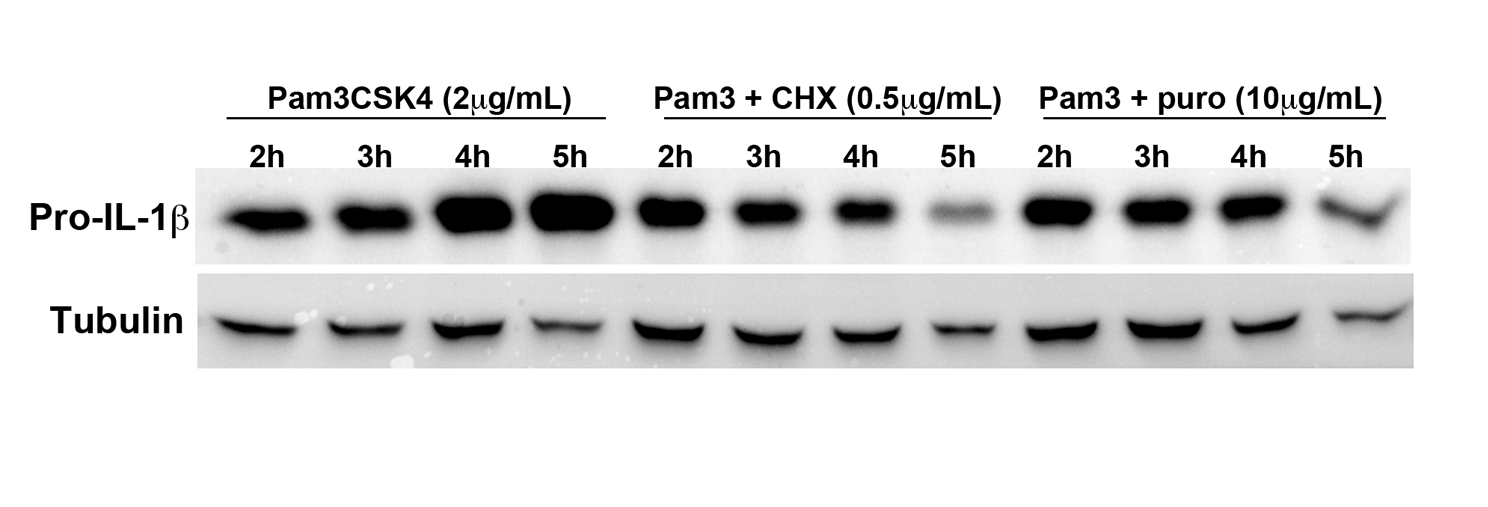

Supplement: Figure S4 — Both cycloheximide and puromycin block IL-1β hyperstimulation. Macrophages were treated with 2 µg/mL Pam3CSK4 for 2 hrs. Cells were then treated with either cycloheximide (0.5 µg/mL) or puromycin for additional 3 hrs and pro- IL-1β protein levels were measured by western blot. (TIF) [file ppat.1004229.s004.tif]

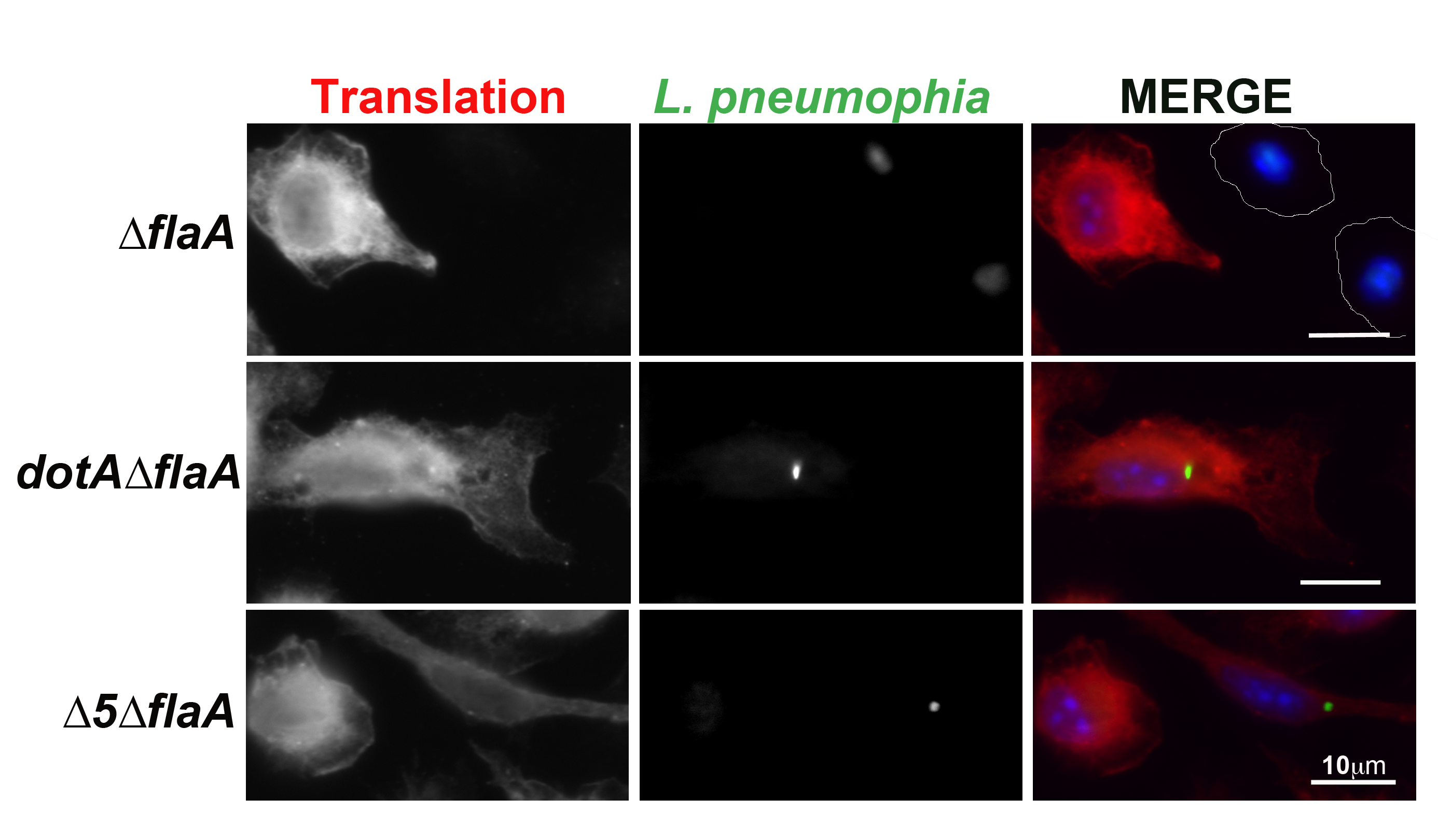

Supplement: Figure S5 — Δ5 mutants show partial inhibition of protein synthesis. B6 macrophages were infected with ΔflaA, dotAΔflaA and Δ5ΔflaA for 5 hrs and 10 µg/mL of puromycin was added between 5–6 hrs poi. Cells were fixed, permeabilized and incorporated puromycin was detected by anti-puromycin antibody (12D10) and fluorescence microscopy. (TIF) [file ppat.1004229.s005.tif]
